# Adolescents’ Experiences of Being a Sibling of a Child With Type 1 Diabetes: A Qualitative Study

**DOI:** 10.1177/26350106261442180

**Published:** 2026-05-20

**Authors:** Ingeborg Enes Ludvigsen, Anne Haugstvedt

**Affiliations:** 1 Western Norway University of Applied Sciences, Bergen, Norway

## Abstract

**Purpose:**

The purpose of the study was to explore adolescents’ experiences of being a sibling of a child with type 1 diabetes (T1D) and how diabetes affects the family.

**Methods:**

A descriptive qualitative study was conducted in Norway through individual, semistructured interviews with adolescents (16-20 years) who had been siblings of a child with T1D before the age of 18. All interviews were audio recorded and transcribed verbatim. Thematic analysis, as described by Braun and Clarke, was used to analyze the data.

**Results:**

Four main themes were identified during the analysis, each comprising related subthemes. The main themes were (1) the start was difficult, but it gradually got better; (2) diabetes affects the atmosphere in the family; (3) a desire to be included; and (4) diabetes affects everyday life to a great extent.

**Conclusions:**

The findings of the study confirmed that T1D has a major impact on a family’s everyday life, but the siblings experienced that it affected the family dynamics both positively and negatively. Siblings expressed a desire for more information and support from health care professionals and a desire to be included in the diabetes management of their sibling with T1D. The findings highlight the need for health care professionals to include siblings in diabetes education and follow-up.

## Introduction

The global prevalence of type 1 diabetes (T1D) in children and adolescents is steadily increasing, and T1D is one of the most common paediatric diseases worldwide, with an estimate of 651 700 children and adolescents living with the condition in 2021.^1^ According to the Norwegian Childhood Diabetes Registry,^2^ Norway has among the highest incidence rates of T1D in children under 15 years old, and 441 children and adolescents ages 0 to 17 were diagnosed with T1D in Norway in 2023.^2^

T1D requires lifelong insulin treatment.^1^ Optimal treatment typically involve intensive insulin therapy through multiple daily injections or continuous subcutaneous insulin infusion.^3^ Automated insulin delivery (AID) systems, integrating insulin pump and continuous glucose monitors (CGMs), are increasingly often used due to their ability to enhance glycaemic control and reduce episodes of hypoglycaemia and hyperglycaemia, particularly at night. These technologies are shown to improve the quality of life for both children with T1D and their caregivers.^4^ However, all these intensive treatment regimens require multiple daily medical decisions and treatment tasks. Thus, insulin treatment is comprehensive and demanding for both the child with T1D and the caregivers.

The daily treatment challenges following T1D affect the entire family to a greater or lesser extent, and the needs of the child with such a chronic disease often become the family’s priority.^5^ Families often experience the initial period following diagnosis as particularly stressful, with uncertainty and lack of knowledge being key factors.^6^ Although families adapt over time, changes in family dynamics, reduced spontaneity, sleep deprivation, and caregiver burden often may persist.^6^

Siblings of children with chronic diseases have reported increased family conflict and tired and exhausted parents.^7^ Some siblings experience injustice in how the parents handle them compared to their sibling with chronic disease, with different rules and expectations.^7^ Siblings often feel responsibility for their brother or sister with chronic disease. The responsibility and involvement are reported as having both positive and negative consequences, with some siblings reporting greater resilience and others a feeling of burden.^6,8,9^ It has also been indicated that siblings desire more information about their siblings’ disease and how they could contribute.^10,11^ and several official documents highlight the importance of acknowledging siblings as next of kin, stating that when a family is affected by a chronic disease in a child.^12,13^ Failure to address the siblings’ needs could negatively affect their emotional well-being, school performance, and social life.^14^

Chan and Shorey^15^ found in their review of qualitative and quantitative research that siblings of children with T1D could experience emotional and behavioural difficulties, lower self-esteem, and feelings of jealousy or unfairness related to the sick child’s special treatment. However, stronger sibling bonds could also develop over time, especially when siblings were involved in caregiving. Fear of disease outcomes and lack of information were key concerns among siblings.^15^ Despite the 10 qualitative studies included in the review of Chan and Shorey,^15^ they highlight the need for more research on this topic. The need for more qualitative research is especially of importance when considering the major changes in the treatment of T1D that have occurred during the recent years, with increased use of CGMs, insulin pumps, and AID systems. These changes have affected the everyday lives of families of a child with T1D. Therefore, the purpose of the present study was to describe adolescents’ experience of having a sibling with T1D and how diabetes affects the family.

## Methods

### Research Design

We used a qualitative descriptive study design to explore the adolescents’ experiences of being siblings of children with T1D. A qualitative descriptive approach is appropriate when straight descriptions of a phenomena are desired.^16^ Thematic analysis as described by Braun and Clarke^17^ was used. Thematic analysis offers the researcher flexibility independent of the theoretical foundation, type of data, and approach.^17^ The method can be applied within a qualitative descriptive design when the focus is on providing a rich, data-near account of participants’ experiences, such as it was in the present study. The Standards for Reporting Qualitative Research checklist was used to ensure quality in reporting and thus increase transparency and credibility.^18^

### Recruitment and Participants

To recruit participants to the study, collaborators in the Norwegian Diabetes Association were contacted. Names of county representatives of the diabetes associations’ family and child committee were received and contacted by phone. County representatives shared information about the study on their platforms where they normally share information with their members, including social media pages. Siblings of children with T1D who were interested in participation were encouraged to contact the researcher.

Initially, adolescents ages 16 to 20 years with more than 1 year experience of being a sibling of a child with T1D were invited to take part in the study. Because few potential participants signed up initially, the age for inclusion was changed so that young adults up to 24 years of age could participate. However, the included participants should have experienced being a sibling of a child with T1D when they were younger than 18 years of age. Adolescents ages 16 to 20 were considered to have sufficient maturity to reflect on the situation they have been or still are in. Adolescents older than 20 years may reflect better despite the fact that there may be some memory-related challenges. The included participants should speak and understand Norwegian language, and siblings <16 years were excluded.

A total of 10 adolescents or their parents showed interest and contacted the researcher. A written information and consent letter was then sent by email to the adolescents. Consent was sent back to the researcher, and the researcher contacted them to make appointments for the interviews. Written informed consent was also obtained from the parents and the siblings with T1D because third-person information about them could be revealed during the interviews even though we focused on the siblings’ experiences. The 10 participants who consented to participate consisted of 4 boys and 6 girls ages 16 to 24 years, with a mean age of 17, and 2 participants were over 20 years old. They came from various counties in Norway, both cities and villages, as described in Table 1.

**Table 1.** Participants and Interview Settings.

Participant	Age	Age of sibling with type 1 diabetes	Years since diagnosis of diabetes in sibling	Interview setting
1 (F)	18	7	2	Zoom
2 (M)	16	12	Participant does not remember	Zoom
3 (F)	20	17	10	Zoom
4 (F)	20	14	6	Zoom
5 (F)	17	14	2	Zoom
6 (M)	18	14	8	Zoom
7 (F)	20	18	11	Zoom
8 (F)	20	18	8	Zoom
9 (M)	24	12	10	Zoom
10 (M)	21	12	10	Telephone

Abbreviations: F, female; M, male.

### Data Collection

A semistructured interview guide was developed by both authors based on the purpose of the study. The purpose of the study and the development of the interview guide was empirically based on perceived existing knowledge gaps and important topics to explore without any specific theoretical or conceptual framework underpinning the study.

During a semistructured interview, it is important to give the participants time and space to talk about their experiences.^19^ The first author (IEL) conducted the interviews, and the interview guide started with introductory questions about the adolescent and their family followed by 4 main questions and follow-up questions. The questions focused on the experiences of being a sibling of a child with T1D, family dynamics, coping, and diabetes follow-up and support from health care professionals. The first 2 interviews were considered as pilot interviews. However, the pilot interviews did not lead to any significant changes in the interview guide; only small adjustments in the follow-up questions were performed. The pilot interviews were therefore included in the analysis. Due to geographical distances, the interviews were conducted digitally via Zoom at a time that suited the participants, and they lasted an average of 34 minutes (range: 20-48). All interviews were audiotaped and transcribed verbatim.

### Data Analysis

The analysis followed the 6 steps of thematic analysis as described by Braun and Clarke,^17^ a flexible method without a specific theoretical foundation, and the analysis team consisted of the 2 authors (IEL and AH). Both authors are experienced nurses. In addition, AH has extensive competence and experience in clinical diabetes and diabetes research, including qualitative methods. An inductive approach that combined semantic and latent levels of analyses were applied. Furthermore, an open-minded and reflective discussion during the analysis took place to avoid any preunderstanding affecting the results.

The 6 steps of the analysis are presented in Table 2. Initially, to become familiar with the data, both authors (researchers) read and reread the transcribed data several times to achieve a good overview of the data material. In phase 2, the authors separately created codes by systematically reviewing the data set to identify parts of the data material that were relevant, interesting, or meaningful for the aim of the study. In phase 3, the authors met for a workshop where the codes from the previous phase were discussed and organized into candidate themes. In the fourth phase, the entire data set was rereviewed to assess whether the candidate themes from the previous phase made sense. The themes that fit were kept, and those that did not were rejected. Furthermore, in phase 5, the authors met for a new workshop and agreed on the essence of the themes and defined and named the final versions. In the final phase, the first author brought the entire analysis process together into a whole and wrote the draft result section of this article.

**Table 2.** The 6 Phases of Thematic Analysis According to Braun and Clarke.^17^

Phases	Description of the phase
1. Familiarizing yourself with the data	Transcribing data, reading and rereading, noting down ideas or insights
2. Coding	Coding interesting segments of the data systematically across the entire data set, collating data relevant to each code
3. Generating initial themes	Collating codes into candidate themes, gathering all coded data relevant to each theme
4. Developing and reviewing themes	Checking that themes make sense in relation to the codes and the full dataset; radical revision is possible if necessary
5. Refining, defining, and naming themes	Fine-tuning the analysis, writing a brief synopsis of each theme, and giving each theme name
6. Writing up	Finishing the analysis and writing process; weaving together the analytic narrative and compelling, vivid data extracts to tell a coherent and persuasive story that addresses the research question

To strengthen the trustworthiness of the study methods in general and the analysis of data specifically, the methods and results were presented and discussed several times with external researchers in an expanded research environment.

### Ethics

Written informed consent was obtained from the participating adolescents. The adolescents were informed that participation was voluntary and of their right to withdraw at any time. They were also informed about publication of the anonymized responses. The researchers (authors) did not have any relationship to the participants or their families. The data material collected was anonymized in accordance with ethical guidelines. The study was conducted in accordance with the Declaration of Helsinki and assessed by the Regional Committee for Medical and Health Research Ethics (REK-vest, ref. 774043), and the Norwegian Centre for Research Data approved the processing of personal data in the study (Sikt, ref. 815406).

## Results

Four main themes were identified regarding the adolescents’ experience of being a sibling of a child with type 1 diabetes: (1) the start was difficult, but it gradually got better; (2) diabetes affects the atmosphere in the family; (3) a desire to be included; and (4) diabetes affects everyday life to a great extent. Within each theme, 2 to 3 subthemes were identified, as shown in Table 3.

**Table 3.** Themes and Subthemes.

Theme	Subtheme
1. The start was difficult, but it gradually got better	(a) Emotional reactions
	(b) Scary experiences related to hypoglycaemia
	(c) Knowledge brings security
2. Diabetes affects the atmosphere in the family	(a) Tired and worried parents are painful to see
	(b) Social restricted parents
	(c) Both positive and negative impact on the family
3. A desire to be included	(a) A feeling of being left out
	(b) A desire of participation in the diabetes management
4. Diabetes affects everyday life to a great extent	(a) Diabetes causes stress and steals attention
	(b) Disturbing alarms, especially at night

## The Start Was Difficult, but It Gradually Got Better

### Emotional Reactions

The interviewed adolescents (Table 1) experienced diabetes in sibling as something completely new and unknown and as something sudden, and the transition to a life with T1D in the family was described as difficult. Several described feelings of anxiety and fear due to not fully understanding what diabetes is. Some described it as scary and that they were worried about their sibling.

I’m going around worrying about my brother with diabetes. It’s a very scary thing, I often go around thinking about it. It was scary when I was growing up, in the sense that it could have quite frightening consequences. (Informant 9)

The informants described adapting to their sibling’s diabetes as a learning process. Over time, as they gained experience, it was described to be easier and that it turned into more of a routine. The interviews also revealed that informants experienced that the parents excused their siblings with diabetes for certain actions because of the diagnosis and that this gave the informants a feeling of injustice. One said:

I kind of remember one time we were at a water park, and he hit me, and I was like, that’s not okay. But then mom and dad were like. “But you have to remember that he has diabetes, and it’s difficult for him right now,” and so on. I kind of had to learn to deal with the fact that he was excused for certain things for a while. (Informant 8)

### Scary Experiences Related to Hypoglycaemia

Some of the informants described unsettling and frightening situations due to low blood glucose in their sibling with diabetes. One had witnessed their sibling having cramps and foaming at the mouth. They explained that such situations caused worries that stayed with them for a long time afterward but also that they learned from them. Overall, they explained feelings of insecurity and uncertainty when dealing with diabetes. Despite some who have received training, several described feeling worried and stressed about the possibility of low blood glucose episodes if they were alone with their sibling. One said:

He had such low blood sugar that he ended up in a sort of coma. He was just lying in bed, shaking and making grimaces and things like that, and I was the one who called the ambulance because dad was outside and didn’t hear mom calling. It was a very scary experience - I thought I was losing my little brother. It stayed with me for a long time afterward. (Informant 3)

### Knowledge Brings Security

Through the interviews, it emerged that the informants desired more knowledge about diabetes because it provided security. They experienced having very little knowledge about diabetes in the beginning, but over time, they gained knowledge and experience that made them feel more secure. One informant described unnecessary worry that could have been avoided with more knowledge. Another described how learning about the use of glucose gel and where it was stored reduced her fear of low glucose levels. Several informants highlighted that their knowledge and experience with diabetes fostered security and an increased understanding in interactions with others affected by diabetes or other chronic diseases.

I tried to measure my blood sugar and my sibling’s blood sugar, and that was a really good learning experience. That way, I knew how to do it if I ever had to help him. It felt reassuring. (Informant 3)

## Diabetes Affects the Atmosphere in the Family

### Tired and Worried Parents Are Painful to See

The informants described that their parents had a lot of responsibility for their sibling with diabetes and the diabetes treatment. Their sleep was affected due to diabetes tasks at night. This led to parents feeling tired and exhausted. One described a mother being burned out due to the diabetes-related distress. The witnessing of their parents’ exhaustion, stress, and worries was difficult for the informants, causing them to feel stressed and anxious as well. One said:

Mom said it was like having a baby again, that it took a lot of her sleep and required a lot of attention and time. (Informant 8)

Another said:

In a way, when you see that both your parents are very worried and stressed, you also become a bit like that, especially when you’re that young. (Informant 7)

### Social Restricted Parents

The informants experienced that diabetes limited their parents’ social lives and personal time. A feeling of sorry for their parents due to these restrictions was described. One said:

I actually feel a bit sorry for them, that they just can’t relax about it, that there are so many things that sort of limit them from being able to go out and have a good time themselves. (Informant 1)

Several of the adolescents explained that close family members and the family’s network lacked diabetes knowledge, making parents anxious about leaving their child in others’ care. This prevented them to take some time off. A situation was described in which a mother withdrew from social life because the child with diabetes only trusted the mother when it came to diabetes treatment.

### Both Positive and Negative Impact on the Family

It was described that diabetes affected the mood and the atmosphere in the family with more arguing and irritation as a result of diabetes. One experienced that parental worries created a tense atmosphere in the family. Several observed that their sibling’s mood fluctuated with the blood glucose level and that their mood affected the entire family. One commented:

So that’s the biggest difference in the dynamics, when people with type 1 diabetes get low or high, you notice it in their mood, you notice it in how they behave. (Informant 6)

On the other hand, some informants felt that diabetes strengthened the family bonds and created a shared experience. One described the family as more united after facing diabetes-related challenges together and said:

It has perhaps brought us together a bit, that it is something we have in common and something we can talk about. (Informant 8)

Several viewed the diabetes community positively, highlighting the Norwegian Diabetes Association’s children and family committee as a valuable support network and the importance of informing families about the Diabetes Association’s offerings for both children with diabetes and their relatives.

## A Desire to Be Included

### A Feeling of Being Left Out

It emerged from the interviews that none of the informants had any responsibility or a specific role in the management of their sibling’s diabetes. Some were fine with not being a helper, while for others, it was difficult. It was also described that siblings with diabetes did not trust or want their help with diabetes care. One said:

I was always left out, I never got involved, I couldn’t do much but I just took everything in. I want to help, I want things to be okay and I want to be able to fix it. But I didn’t get to do much. It was really uncomfortable. (Informant 4)

Several adolescents described a feeling that the sibling with diabetes was prioritized in the family, often leaving them with a feeling of being down-prioritized, although they in a way, understood why. They described their sibling’s needs always coming first while their own needs came second. Another felt left to fend on their own and to take care of things alone. One described:

You kind of feel like they are prioritized quite a bit over yourself, that you’re somewhat put aside. He is always first, my needs come later. (Informant 7)

### A Desire of Participation in the Diabetes Management

To feel more confident and understand their sibling’s diabetes, the informants described a desire to be more included. One said:

I really wish that they would see us a bit more, like, that we are a part of it too, and that we are also affected by it. So maybe we could get a bit more follow-up by health care professionals, like what it is and how it works, and that we could feel more included in the treatment of it. (Informant 1)

The adolescents expressed a desire to be seen as part of the diabetes care and how diabetes affects them also. Several wanted more information early on from health care professionals in order to feel more involved. They suggested that the health care professionals should inform siblings about diabetes and what to expect in the first period after their sibling was diagnosed and be available for the sibling and address them as relatives. This would have given them a sense of security.

Some informants mentioned they had been shown a child-friendly and easy understandable video at the hospital about diabetes that helped them better understand diabetes. None of the informants described having received training on diabetes management from health care professionals, although some wanted such training to ease the burden on their parents. Some described that they learned how to measure blood glucose and administrate insulin from their parents despite not having a special role in diabetes care. Others learned by observing what the parents did or figuring things out themselves. One said:

I kind of learned what to do because I saw what mom and dad did, so I have watched and learned it from mom and dad. (Informant 5)

## Diabetes Mostly Affects Everyday Life

### Diabetes Causes Stress and Steals Attention

The informants felt that their sibling’s diabetes took up much of their parents’ attention, often regardless of the reasons. Several thought it was unfair that their sibling received more focus than them. One informant described feeling that their parents’ focus was on the sibling with diabetes, which led the informant’s experience of having to take responsibility for raising themselves. Some understood why their sibling received more attention, while others did not fully grasp it until they were older.

Now that I’m older, it’s fine because I understand that she doesn’t have a choice, she needed to have that attention. But I remember thinking it was unfair that she got so much attention. (Informant 5)

The informants noted that diabetes management before meals often took focus and was time-consuming, especially the insulin administration. Because of waiting, one described that the food could often get cold. This could easily lead to irritation. Several described their siblings’ diabetes as a constant thing the family always had to think about and which was always brought up and talked about in daily life. Whether going out or on vacation, the family had to plan around diabetes. One described a separate suitcase for diabetes equipment on vacation. Diabetes was experienced as a source of stress for the family.

No, the first period I would describe as very stressful, really stressful actually. There was so much uncertainty. My family was calmer before my brother got this disease, so it became a constant stress factor that was always there. (Informant 9)

### Disturbing Alarms, Especially at Night

Through the interviews, it emerged that several informants found alarms from their sibling’s diabetes equipment disturbing. The beeping caused stress, disturbed their sleep, and made them worry about their sibling.

If we heard beeping from the pump at night, it’s a bit stressful wondering if everything is okay and if mom is taking care of it. (Informant 10)

It was mostly the parents who handled the alarms because few of the informants were allowed to help their sibling. However, some experienced that they had to notify their parents about the alarms because they occasionally shared room with their sibling.

## Discussion

The purpose of the study was to explore adolescents’ experiences of being siblings to children with T1D and how this affects the family. The main findings showed that the informants experienced various negative emotions related to their siblings being diagnosed but that over time, they gained knowledge and experience, something that increased their confidence and understanding. Diabetes affected everyday life and family dynamics both positively and negatively. The sibling informants without diabetes often had a feeling of being left out in the family, and they described a desire to be more included in the diabetes management. By highlighting siblings’ perspectives, this study may contribute to a better understanding of siblings’ needs and how health care professionals should provide better support to them and the entire family.

The study findings highlight that siblings of children with T1D often experience their sibling’s diabetes as a constant thing the family always had to think about in everyday life. Many found it particularly difficult to see their parents exhausted and socially restricted due to the diabetes management. Also, a study of Schuman et al^7^ found that siblings of children with chronic illnesses often reported tired and exhausted parents and increased family conflicts. Furthermore, Baker and Claridge^6^ indicated that mothers described feeling restricted and rarely had an opportunity to go out alone with their partner due to their child’s chronic illness. In addition to a restricted social life, the informants also highlight how diabetes significantly impacted the sleep patterns within the family. Reduced sleep due to treatment and observation of the child’s chronic illness was also reported by Baker and Claridge.^6^ In addition to disrupted sleep among the parents, informants described that their own sleep also was affected. They were awakened by alarms from their sibling’s diabetes equipment at night. This kept them awake and caused worries and stress for some, as they lay listening to the alarm and their parents’ activity without fully understanding what was happening. However, diabetes could also have a positive impact on the family. Some of the informants described strengthened family bonds and shared experiences. Also, Baker and Claridge^6^ reported that some families experienced stronger cooperation and communication when coping with chronic illness.

In the present study, the informants described how their sibling’s diabetes demanded much of their parents’ attention, leading to feelings of being left to fend on their own and feeling deprioritized. This finding is supported by previous research showing that siblings of children with chronic illnesses often experience receiving less attention from their parents due to their parents focusing on the chronically ill child.^5,7,8,15^ While some of the informants understood why their siblings received more attention, others did not fully grasp it until they were older. Chan and Shorey^15^ described how siblings gradually over time develop an understanding of their parents’ focus on their sibling with T1D. Consequently, Blamires et al^10^ highlighted in their study that siblings of children with chronic illnesses often develop greater independence and maturity compared to peers.

None of the informants in the study described having a significant role or responsibility in the diabetes management of their sibling. Some were comfortable with this, while others found it difficult due to the feeling of being left out. In contrast, previous research has shown that siblings often are involved in the management of chronic illness in children.^8,9,15^ Furthermore, Kelada et al^8^ found that siblings involved in caregiving tasks for a chronically ill sibling reported less concerns and better sibling relationships and demonstrated greater use of problem-focused coping compared to those not involved. Similarly, Lummer-Aikey and Goldstein^9^ found that some siblings perceived being a helper as positive, facilitating better adaption to the illness. Accordingly, the informants in the present study described feelings of anxiety and fear due to not fully understanding the diabetes treatment and treatment tasks. However, the findings indicate that not all siblings want an active role. Some were fine with not having any responsibility, as also described by Lummer-Aikey and Goldstein.^9^ For some siblings, being a helper and having responsibility for their chronically ill sibling could be experienced as a burden.^9^ Overall, both previous research and findings in the present study highlight that siblings of children with chronic illnesses have different needs and preferences regarding involvement. This underscores the importance of recognizing siblings as next of kin and supporting their individual needs in diabetes education and clinical diabetes follow-up. However, more research is needed, both more studies of siblings’ experiences in an era of modern technological diabetes treatment and studies that examine how siblings’ role is experienced by parents and the children with diabetes themselves. And not least, there is a need for intervention studies that test different models for education and follow-up of families where a child has T1D, interventions that also include siblings.

### Strengths and Limitations

This study has both strengths and limitations, which are considered in light of the concept of trustworthiness. In accordance with Lincoln and Guba,^20^ a qualitative study’s trustworthiness depends on credibility, dependability, confirmability, and transferability. The research team’s experience and knowledge of the studied phenomenon may have strengthened the study’s credibility by helping the researchers to collect rich and sufficient data. To avoid that the preunderstanding of the phenomenon should limit the interpretation of the data, the study results were discussed with external researchers several times. The researchers had no prior relationship with the adolescents interviewed or their families. Dependability was maintained through a well-described design and research process. A well-described analysis process and reflexive and open-minded discussions of data strengthen the study’s confirmability. Although a relatively small sample, the diversity of the sample and the rich data along with the quotations and detailed descriptions strengthen the study’s transferability. However, self-recruitment may have led to selection bias, with primarily the most engaged individuals choosing to participate. Finally, member checking could have strengthened the study trustworthiness and credibility. However, this was not conducted because the participants were a young and potentially vulnerable group that was difficult to recruit. Recontacting them was therefore considered inappropriate and potentially burdensome.

## Conclusion

The findings of the study confirmed that T1D has a major impact on a family’s everyday life, but the siblings experienced that it affected the family dynamics both positively and negatively. Siblings expressed a desire for more information and support from health care professionals and a desire to be included in the diabetes management of their sibling with T1D. The findings highlight the need for health care professionals to recognize and address the needs of children as next of kin and include siblings in diabetes education and follow-up.
